# Neurobiology of Alcohol Dependence: Focus on Motivational Mechanisms

**Published:** 2008

**Authors:** Nicholas W. Gilpin, George F. Koob

**Keywords:** Alcoholism, alcohol dependence, chronic alcohol and other drug effect (AODE), neurology, brain, animal models, animal studies, neurodegeneration, neuroadaptation, positive reinforcement, negative reinforcement, neural circuits, neurotransmitter, dopamine, opoids, γ-aminobutyric acid (GABA), glutamate, serotonin

## Abstract

Alcoholism is a debilitating disorder for the individual and very costly for society. A major goal of alcohol research is to understand the neural underpinnings associated with the transition from alcohol use to alcohol dependence. Positive reinforcement is important in the early stages of alcohol use and abuse. Negative reinforcement can be important early in alcohol use by people self-medicating coexisting affective disorders, but its role likely increases following the transition to dependence. Chronic exposure to alcohol induces changes in neural circuits that control motivational processes, including arousal, reward, and stress. These changes affect systems utilizing the signaling molecules dopamine, opioid peptides, γ-aminobutyric acid, glutamate, and serotonin, as well as systems modulating the brain’s stress response. These neuroadaptations produce changes in sensitivity to alcohol’s effects following repeated exposure (i.e., sensitization and tolerance) and a withdrawal state following discontinuation of alcohol use. Chronic alcohol exposure also results in persistent neural deficits, some of which may fully recover following extended periods of abstinence. However, the organism remains susceptible to relapse, even after long periods of abstinence. Recent research focusing on brain arousal, reward, and stress systems is accelerating our understanding of the components of alcohol dependence and contributing to the development of new treatment strategies.

Alcoholism, also called dependence on alcohol, is a chronic relapsing disorder that is progressive and has serious detrimental health outcomes. The development of alcoholism is characterized by frequent episodes of intoxication, preoccupation with alcohol, use of alcohol despite adverse consequences, compulsion to seek and consume alcohol, loss of control in limiting alcohol intake, and emergence of a negative emotional state in the absence of the drug (American Psychiatric Association 1994). According to the National Institute on Alcohol Abuse and Alcoholism (NIAAA), more than 17 million people in the United States either abuse or are dependent on alcohol (NIAAA 2007*a*), with a cost to U.S. society of over $180 billion annually (NIAAA 2004*a*).

A major goal of basic research on alcoholism is to understand the neural underpinnings of alcohol use and the pathological progression to alcohol dependence (NIAAA 2007*b*). Preclinical research uses a wide array of techniques to assess the molecular, cellular, and behavioral events associated with the transition to alcohol dependence. These techniques are used in conjunction with animal models that mimic various components of alcohol dependence in humans. Chronic exposure to high doses of alcohol produces counteradaptive neural changes that affect the motivational properties of alcohol and drive subsequent alcohol-seeking behavior.

This article summarizes some basic neurobiological mechanisms associated with the motivational aspects of alcohol dependence. It briefly discusses the roles of brain reward and stress systems in alcohol dependence. Additionally, the article reviews the late stages of alcohol dependence, specifically the neurodegeneration associated with long-term alcohol exposure and the potential for relapse and recovery during long-term abstinence.

## Reinforcement and the Transition From Alcohol Use to Dependence

Reinforcement is a process in which a response or behavior is strengthened based on previous experiences. Positive reinforcement describes a situation in which a presumably rewarding stimulus or experience (e.g., alcohol-induced euphoria) increases the probability that the individual exhibits a certain response (e.g., alcohol-seeking behavior). Negative reinforcement occurs when the probability of an instrumental response (e.g., alcohol-seeking behavior) increases if this response allows the individual to circumvent (i.e., avoidance response) or alleviate (i.e., escape response) an aversive stimulus. In alcohol dependence, the aversive stimulus often is composed of motivational/affective symptoms (e.g., anxiety, dysphoria, irritability, and emotional pain) that manifest in the absence of alcohol (i.e., during withdrawal) and which result from prior discontinuation of alcohol consumption. Thus, people may drink to prevent or alleviate the anxiety they experience during alcohol withdrawal. In conditioned positive and negative reinforcement, stimuli that become associated with either alcohol or withdrawal can motivate subsequent alcohol-seeking behavior. Alcohol-drinking behavior is driven by both positive and negative reinforcement, although their relative contributions change during the transition from alcohol use to abuse to dependence.

One approach for the study of reinforcement in animal models of alcoholism is a procedure called operant conditioning. With this approach, animals are trained to perform a response (e.g., press a lever or nose-poke a hole) that results in delivery of a stimulus (e.g., a small amount of alcohol) that animals are motivated to obtain. Operant conditioning procedures can be fine-tuned to include different work requirements for stimuli with varying degrees of motivational value for the individual tested. This procedure models how humans exhibit varying degrees of willingness to work for alcohol and other drugs under many different conditions.

### Positive Reinforcement

The positive reinforcing effects of alcohol generally are accepted as important motivating factors in alcohol-drinking behavior in the early stages of alcohol use and abuse. These effects most often are examined using animal models of self-administration. With different operant conditioning procedures, researchers can determine the time course, pattern, and frequency of responding for alcohol. For example, investigators can use progressive-ratio schedules of reinforcement, in which the number of responses (e.g., lever presses) required for subsequent delivery of the reinforcer (e.g., alcohol) gradually increases throughout a session. This procedure allows researchers to determine the maximum number of responses (i.e., the breakpoint) that animals are willing to perform to obtain a single reinforcer. Operant procedures most often are used to examine oral self-administration of alcohol, but they also can be used to assess self-administration of alcohol via other routes. For example, rats will respond for alcohol infusions directly into the stomach (Fidler et al. 2006), blood stream (Grupp 1981), or brain (Gatto et al. 1994).

An alternative to operant procedures, free-choice responding allows researchers to examine alcohol consumption and preference in rats in their home-cage environment. In this procedure, alcohol is available to the animals via normal drinking bottles in the home cage. Free-choice procedures incorporate a variety of experimental manipulations, such as offering multiple bottles with different alcohol concentrations, varying the schedules of when and for how long alcohol is available, and adding flavorants to available solutions. These manipulations provide valuable additional information about the preference for alcohol.

Another method for assessing the reinforcing properties of alcohol is intracranial self-stimulation (ICSS). In this procedure, rats are implanted with electrodes in discrete brain regions and then are allowed to self-administer mild electrical shocks to those regions via standard operant procedures. Rats readily self-administer shocks to brain regions that are important in mediating the rewarding properties of alcohol. The strength of the electrical stimulation needed for the animal to maintain responding reflects the reward value of the ICSS. Thus, if only mild electrical stimulation of a certain brain region is required to maintain responding, ICSS is said to have a high reward value; if, by contrast, a stronger electrical stimulation of a given brain region is required, then ICSS is said to have a lower reward value. Alcohol increases the reward value of ICSS because in the presence of alcohol, weaker electrical stimulation is required to maintain responding (e.g., Lewis and June 1990).

Finally, the reinforcing properties of alcohol can be assessed using a procedure called place conditioning. In this procedure, contextual cues repeatedly are associated with the presence or absence of alcohol availability—for example, rodents repeatedly are placed in a specific environment where they receive alcohol and in another environment where they receive no alcohol. Subsequently, the rodents are allowed to freely explore these environments. If the animal spends more time exploring the alcohol-paired environment, then this behavior is hypothesized to reflect the conditioned positive reinforcing effects of alcohol. Interestingly, this procedure is used primarily with mice because they exhibit a preference for alcohol-paired environments, whereas rats typically exhibit an aversion to environments that previously were paired with alcohol (Cunningham et al. 1993).

Some recently developed animal models mimic binge drinking in humans. This pattern of self-administration, defined in humans as an excessive pattern of alcohol drinking that produces blood alcohol levels greater than 0.08 percent within a 2-hour period, may be associated with dependence (NIAAA 2004*b*). Models of binge drinking have been developed for both adult (Ji et al. 2008) and adolescent (Truxell et al. 2007) rats and intend to mimic drinking behavior motivated primarily by the positive reinforcing effects of alcohol early in the transition to dependence. For example, sweeteners often are added to the alcohol solution in these models, a procedure that is thought to reflect the situation in humans because people tend to begin drinking alcohol in sweetened beverages (Gilbert 1978; Samson et al. 1996). Other approaches successfully have used genetic selection to produce animals that readily self-administer alcohol in a binge-like pattern (e.g., Grahame et al. 1999; Lumeng et al. 1977). For this approach, high-and low-alcohol–drinking rodents are selectively bred over generations to produce lines that are either highly prone or highly resistant to voluntary alcohol consumption. The resultant phenotypes^1^ may provide models of specific subtypes of alcoholism.

### Negative Reinforcement

As mentioned above, the early stages of alcohol use and abuse mainly are associated with alcohol’s positive reinforcing effects. However, alcohol’s negative reinforcing effects may contribute to alcohol-drinking behavior at this stage in people who suffer from coexisting psychiatric disorders and use alcohol to self-medicate these disorders. Comorbidity of alcohol problems (i.e., abuse or dependence) with anxiety and depressive/bipolar disorders is high (44 percent and 50 percent, respectively) (Kushner et al. 1990; Weissman et al. 1980). Thus, these people may use alcohol to alleviate the symptoms of the coexisting disorders. Generally, however, the negative reinforcing effects of alcohol become a critical component of the motivation to drink alcohol during the transition to dependence, when withdrawal symptoms occur following discontinuation of alcohol use and the individual drinks to avoid those withdrawal symptoms.

In animal models, the negative reinforcing properties of alcohol often are studied during periods of imposed abstinence after chronic exposure to high doses of alcohol. Such studies have identified an alcohol deprivation effect—that is, a transient increase in alcohol-drinking behavior following long-term alcohol access and a period of imposed abstinence (Sinclair and Senter 1967). Similarly, chronic inhalation of alcohol vapor can reliably produce large elevations in alcohol self-administration (Roberts et al. 1996, 2000*a*), an effect that is amplified when animals repeatedly are withdrawn from the alcohol vapor (O’Dell et al. 2004) and which lasts well into protracted abstinence (Gilpin et al. 2008*b*). Moreover, researchers can use nutritionally complete, alcohol-containing liquid diets to induce alcohol dependence (Frye et al. 1981). Again, symptoms of dependence are augmented when animals repeatedly are withdrawn from the alcohol diet (Overstreet et al. 2002). In general, studies using these approaches have demonstrated that the pattern of alcohol exposure (i.e., the frequency of withdrawals) appears to be as important as the cumulative alcohol dose in revealing alcohol’s negative reinforcing properties.

In addition to these approaches, the negative reinforcing effects of alcohol can be examined using all the models described above (see the section entitled “Positive Reinforcement”), except that testing occurs during imposed withdrawal/abstinence from alcohol. For example, alcohol withdrawal decreases the reward value of ICSS because the threshold of electrical stimulation required to maintain responding is increased (Schulteis et al. 1995).

### Neuroadaptation

Changes in the reinforcing value of alcohol during the transition from alcohol use and abuse to dependence reflect (counter)adaptive neural changes resulting from chronic exposure to high alcohol doses. As stated above, during the early stages of nondependent alcohol use, drinking behavior largely is motivated by alcohol’s positive reinforcing effects, whereas in the dependent state it likely is driven by both the positive and negative reinforcing effects of the drug. Multiple processes contribute to the increased motivation to seek drugs during the development of dependence. Sensitization refers to an increase in the reinforcement value of drugs following repeated exposures. Tolerance refers to a decrease in the reinforcing efficacy of drugs following repeated exposures. Once neuroadaptation has occurred, removal of alcohol from the organism leads to a withdrawal syndrome.

#### Sensitization

The incentive sensitization theory of addiction posits that addictive drugs activate a common neural system responsible for attributing incentive salience to events and stimuli associated with the activation of that system (Robinson and Berridge 1993). As a result, the “liking” of alcohol’s effects becomes closely associated with “wanting” the alcohol-associated incentive stimuli. Following repeated drug exposure, this wanting becomes stronger and transforms into pathological craving for the drug. The incentive sensitization theory of addiction most often is used to describe dependence on addictive drugs other than alcohol. However, alcohol stimulates locomotor activity in mice, an effect thought to correspond to alcohol-induced euphoria in humans, and mice become sensitized to this effect following repeated alcohol exposure (Phillips and Shen 1996), suggesting a role for sensitization in the development of alcohol dependence. This seems to be a species-specific effect, however, because alcohol-induced stimulation of locomotor activity rarely is observed in rats, and little evidence suggests that sensitization to this effect occurs in these animals.

#### Tolerance

An organism that is chronically exposed to alcohol develops tolerance to its functional (e.g., motor-impairing) effects (LeBlanc et al. 1975), metabolic effects (Wood and Laverty 1979), and reinforcing properties (Walker and Koob 2007). Once tolerance to the pleasurable (i.e., hedonic) effects of alcohol develops, the individual requires gradually higher doses of alcohol to produce the same effect previously experienced at lower doses. In animal experiments, this process is reflected by the fact that the animal will work harder to obtain alcohol on a progressive-ratio schedule. In a cyclical pattern, these gradually increasing alcohol doses produce even more tolerance to the hedonic effects of alcohol. Moreover, the clearance of alcohol from the body of an individual with high tolerance can produce a withdrawal syndrome defined by symptoms that are largely the opposite of the effects of alcohol itself.

#### Withdrawal

Following chronic alcohol exposure, the removal of alcohol reliably produces a constellation of withdrawal symptoms, some of which increase the motivation to seek and ingest alcohol (i.e., have motivational significance). Although alcohol withdrawal symptoms vary in severity according to the history of the individual, they are qualitatively similar across species. The physiological aspects of withdrawal in humans and rodents usually last up to 48 hours following termination of alcohol exposure and include convulsions, motor abnormalities, and autonomic disturbances (e.g., sweating, higher heart rate, and restlessness) (Isbell et al. 1955; Majchrowicz 1975). Additionally, withdrawal is associated with a negative-affective state characterized by anxiety, dysphoria, and irritability that typically develops during early stages of withdrawal but can be very long lasting. Perhaps the most reliable of these disturbances across species is an increase in anxiety (Hershon 1977; Valdez et al. 2002). The allostasis theory of alcoholism posits that the negative-affective state produced by this withdrawal state is a major driving force in the propensity for relapse to alcohol-seeking behavior (Koob 2003).

## Brain Circuits Mediating Alcohol Reinforcement

**Figure 1:**
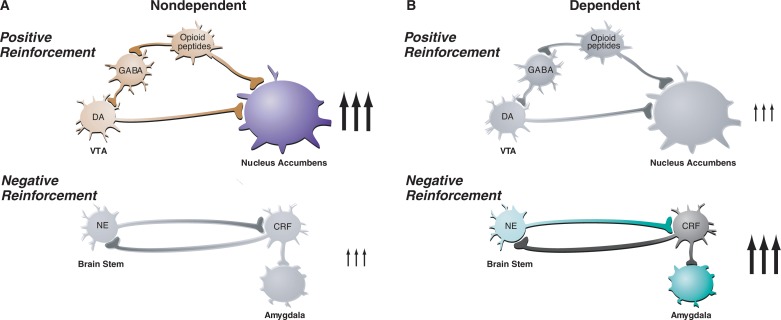
Changes in the activity of the reward circuit mediating the acute positive reinforcing effects of alcohol and the stress circuit mediating negative reinforcement of dependence during the transition from nondependent alcohol drinking to dependent drinking. Key elements of the reward circuit are dopamine (DA) and opioid peptide neurons that act at both the ventral tegmental area (VTA) and the nucleus accumbens and which are activated during initial alcohol use and early stages of the progression to dependence (i.e., the binge/intoxication stage). Key elements of the stress circuit are corticotropin-releasing factor (CRF) and norepinephrine (NE)-releasing neurons that converge on γ-aminobutyric acid (GABA) interneurons in the central nucleus of the amygdala and which are activated during the development of dependence. SOURCE: Modified with permission from Nestler 2005.

A neural circuit can be conceptualized as a series of nerve cells (i.e., neurons) that are interconnected and relay information related to a specific function. Within such a circuit, information is passed between neurons via electrochemical signaling processes. Activated neurons release chemical signaling molecules (i.e., neurotransmitters) that bind to specific proteins (i.e., receptors) on other neurons. Depending on the neurotransmitter involved, this binding leads to the electrical excitation or inhibition of subsequent neurons in the circuit. (For more information on nerve signal transmission, neurotransmitters, and their receptors, see the article by Lovinger, pp. 196–214.) Alcohol interacts with several neurotransmitter systems in the brain’s reward and stress circuits. These interactions produce alcohol’s acute reinforcing effects. Following chronic exposure, these interactions result in changes in neuronal function that underlie the development of sensitization, tolerance, withdrawal, and dependence. Research using pharmacological, cellular, molecular, imaging, genetic, and proteomic techniques already has elucidated details of some of these alcohol effects, and some of these findings will be discussed in other articles in this and the companion issue of *Alcohol Research & Health*. As a foundation for this discussion, the following sections briefly introduce some of the neural circuits relevant to alcohol dependence, categorized by neurotransmitter systems; however, this discussion is by no means exhaustive. Figure 1 illustrates the changing role of positive and negative reinforcement circuits during the transition from the nondependent to the dependent state. The table summarizes the effects of interventions with these signaling systems on various aspects of positive and negative reinforcement.

### Reward Circuits and Neurotransmitter Systems

#### Dopamine Systems

Dopamine is a neurotransmitter primarily involved in a circuit called the mesolimbic system, which projects from the brain’s ventral tegmental area to the nucleus accumbens. This circuit influences how organisms orient toward incentive changes in the environment—that is, it affects incentive motivation.^2^ Studies suggest that dopamine also has a role in the incentive motivation associated with acute alcohol intoxication. For example, alcohol consumption can be blocked by injecting low doses of a compound that interferes with dopamine’s normal activity (i.e., a dopamine antagonist) directly into the nucleus accumbens (Hodge et al. 1997; Rassnick et al. 1992). Furthermore, alcohol ingestion and even the anticipation that alcohol will be available produce dopamine release in the nucleus accumbens as determined by increased dopamine levels in the fluid outside neurons (Weiss et al. 1993). However, lesions of the mesolimbic dopamine system do not completely abolish alcohol-reinforced behavior, indicating that dopamine is an important, but not essential, component of alcohol reinforcement (Rassnick et al. 1993). Finally, alcohol withdrawal produces decreases in dopamine function in dependent individuals, and this decreased dopamine function may contribute to withdrawal symptoms and alcohol relapse (Melis et al. 2005; Volkow et al. 2007).

#### Opioid Systems

Endogenous opioids are small molecules naturally produced in the body that resemble morphine and have long been implicated in the actions of opiate drugs and alcohol. There are three classes of endogenous opioids: endorphins, enkephalins, and dynorphins. They all exert their effects by interacting with three subtypes of opioid receptors—μ, δ, and κ. Researchers have hypothesized that positive alcohol reinforcement is mediated at least in part by the release of endogenous opioids in the brain. This hypothesis is supported by numerous studies demonstrating that opioid antagonists acting either at all opioid receptor subtypes or only at specific subtypes suppress alcohol drinking in a variety of species and models (for a review, see Ulm et al. 1995). Moreover, complete inactivation (i.e., knockout) of the μ-opioid receptor blocks alcohol self-administration in mice (Roberts et al. 2000*b*). The agent naltrexone, a subtype-nonspecific opioid receptor antagonist, currently is approved as a treatment for alcoholism in humans and is particularly effective in reducing heavy drinking.

**Figure 2:**
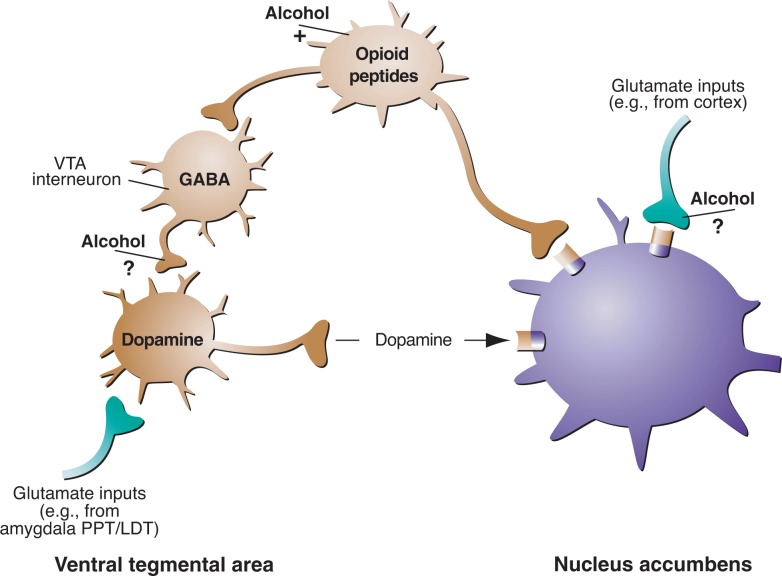
Alcohol’s effects on neurotransmitter systems involved in the brain’s reward pathways. Alcohol, by promoting γ-aminobutyric acid (GABA) subtype GABA_A_ receptor function, may inhibit GABAergic transmission in the ventral tegmental area (VTA), thereby disinhibiting (i.e., activating) VTA dopamine. As a result, these neurons release dopamine in the nucleus accumbens, activating reward processes there. Similarly, alcohol may inhibit release of the excitatory neurotransmitter glutamate from nerve terminals that act on neurons in the nucleus accumbens. Many additional mechanisms (not shown) are proposed, through which alcohol may act on these pathways. Some evidence suggests that alcohol may activate endogenous opioid pathways and possibly endogenous cannabinoid pathways (not shown). NOTE: PPT/LDT, peduncular pontine tegmentum/lateral dorsal tegmentum. SOURCE: Modified with permission from Nestler 2005.

Opioid systems influence alcohol drinking behavior both via interaction with the mesolimbic dopamine system and also independent of the mesolimbic dopamine system, as demonstrated by alcohol-induced increases in extracellular endorphin content in the nucleus accumbens (see figure 2) (Olive et al. 2001). Opioid receptor antagonists interfere with alcohol’s rewarding effects by acting on sites in the ventral tegmental area, nucleus accumbens, and central nucleus of the amygdala (Koob 2003).

#### γ-Aminobutyric Acid Systems

γ-Aminobutyric acid (GABA) is the major inhibitory neurotransmitter in the brain. It acts via two receptor subtypes called GABA_A_ and GABA_B_. Alcohol can increase GABA activity in the brain through two general mechanisms:

- It can act on the GABA-releasing (i.e., presynaptic) neuron, resulting in increased GABA release; or
- It can act on the signal-receiving (i.e., postsynaptic) neuron, facilitating the activity of the GABA_A_ receptor.

Alcohol drinking is suppressed by compounds that interfere with the actions of the GABA_A_ receptor (i.e., GABA_A_ receptor antagonists) as well as compounds that stimulate the GABA_B_ receptor (i.e., GABA_B_ agonists) in the nucleus accumbens, ventral pallidum, bed nucleus of the stria terminalis, and amygdala (for a review, see Koob 2004). Of these, the central nucleus of the amygdala—a brain region important in the regulation of emotional states—is particularly sensitive to suppression of alcohol drinking by compounds that act on the GABA systems (i.e., GABAergic compounds) (Hyytia and Koob 1995). Indeed, acute and chronic alcohol exposure produce increases in GABA transmission in this brain region (Roberto et al. 2003, 2004*a*). Additionally, compounds that target a specific component of the GABA_A_ receptor complex (i.e., the α_1_-subunit)^3^ suppress alcohol drinking when they are injected into the ventral pallidum, an important region that receives signals from neurons located in the extended amygdala (Harvey et al. 2002; June et al. 2003).

Chronic alcohol exposure also leads to alterations in the GABA systems. For example, in some brain regions, alcohol affects the expression of genes that encode components of the GABA_A_ receptor. This has been demonstrated by changes in the subunit composition of the receptor in those regions, the most consistent of which are decreases in α_1_-and increases in α_4_-subunits (for a summary, see Biggio et al. 2007).

The function of GABA_A_ receptors also is regulated by molecules known as neuroactive steroids (Lambert et al. 2001) that are produced both in the brain and in other organs (i.e., in the periphery). Alcohol increases the brain levels of many neuroactive steroids (Van Doren et al. 2000). This increased activity of neuroactive steroids in the brain following alcohol exposure is not dependent on their production by peripheral organs (Sanna et al. 2004). Together, these findings suggest that neuroactive steroids are potential key modulators of altered GABA function during the development of alcohol dependence, perhaps by acting directly at GABA_A_ receptors (Sanna et al. 2004).

#### Glutamate Systems

Glutamate is the major excitatory neurotransmitter in the brain; it exerts its effects via several receptor subtypes, including one called the *N*-methyl-d-aspartate (NMDA) receptor. Glutamate systems have long been implicated in the acute reinforcing actions of alcohol, and alcohol effects perceived by an organism can be mimicked with NMDA receptor antagonists (Colombo and Grant 1992). In contrast to its effects on GABA, alcohol inhibits glutamate activity in the brain. For example, acute alcohol exposure reduces extracellular glutamate levels in a brain region called the striatum, which contains the nucleus accumbens, among other structures (Carboni et al. 1993). Acute alcohol administration also suppresses glutamate-mediated signal transmission in the central nucleus of the amygdala, an effect that is enhanced following chronic alcohol exposure (Roberto et al. 2004*b*). Alcohol affects glutamate transmission most likely by altering the functions of both NMDA receptors (Lovinger et al. 1989) and another receptor subtype known as metabotropic glutamate subtype 5 receptors (mGluR5) (Blednov and Harris 2008). The involvement of NMDA receptors in alcoholism is especially interesting because they also play a role in neuroplasticity, a process characterized by neural reorganization that likely contributes to hyperexcitability and craving during alcohol withdrawal^4^ (Pulvirenti and Diana 2001).

Compounds targeting the glutamate systems also are being used in the treatment of alcohol dependence. For example, the agent acamprosate modulates glutamate transmission by acting on NMDA and/or metabotropic glutamate receptors (for a review, see Littleton 2007). Thus, by dampening excessive glutamate activity, acamprosate blocks excessive alcohol consumption. This process appears to depend on the involvement of genes such as *Per2*, which typically is involved in maintaining the normal daily rhythm (i.e., the circadian clock) of an organism (Spanagel et al. 2005). Acamprosate’s ability to suppress alcohol drinking has been observed across species, and the drug has been approved for the treatment of alcoholism in humans, primarily for its perceived ability to reduce alcohol craving and negative affect in abstinent alcoholics (Littleton 2007).

#### Serotonin Systems

The neurotransmitter serotonin (also known as 5-hydroxytryptamine or 5-HT) has long been a target of interest for potential pharmacotherapies for alcoholism because of the well-established link between serotonin depletion, impulsivity, and alcohol-drinking behavior in rats and humans (Myers and Veale 1968; Virkkunen and Linnoila 1990). Pharmacological compounds that target the serotonin system by inhibiting neuronal reuptake of serotonin,^5^ thereby prolonging its actions, or by blocking specific serotonin receptor subtypes have been shown to suppress alcohol-reinforced behavior in rats (for a review, see Johnson 2008). However, some researchers are debating whether these compounds can affect alcohol-reinforced behavior without affecting consummatory behavior in general. During alcohol withdrawal, serotonin release in the nucleus accumbens of rats is suppressed, and this reduction is partially reversed by self-administration of alcohol during withdrawal (Weiss et al. 1996).

### Stress Circuits and Neurotransmitter Systems

#### Corticotropin-Releasing Factor and Neuropeptide-Y Systems

Recent research has led to the hypothesis that the transition to alcohol dependence involves the dysregulation not only of neural circuits involved in reward but also of circuits that mediate behavioral responses to stressors. Alcohol-induced perturbation of the brain’s stress and antistress systems contributes to the negative emotional state characteristic of alcohol withdrawal. One stress system involves the signaling molecule corticotropin-releasing factor (CRF). CRF produced in and released from the hypothalamus activates the body’s major stress system, called the hypothalamic–pituitary–adrenal (HPA) axis. However, activation of extrahypothalamic CRF systems also produces high anxiety-like states in animals. Several observations indicate that extrahypothalamic CRF contributes to the development of alcohol dependence. For example, alcohol-dependent rats exhibit increased extracellular CRF content in the central nucleus of the amygdala (Merlo-Pich et al. 1995). Moreover, CRF antagonists injected directly into this brain structure suppress both the anxiety-like behavior (Rassnick et al. 1993) and the increase in alcohol drinking (Funk et al. 2006) that are associated with alcohol dependence.

Another molecule involved in regulating the body’s stress response is called neuropeptide-Y (NPY). It has a neural and behavioral profile that in almost every aspect is opposite to that of CRF. For example, NPY has powerful anxiety-reducing effects in animals. Moreover, alcohol-dependent rats exhibit decreased NPY content in the central nucleus of the amygdala during withdrawal (Roy and Pandey 2002), whereas, as stated above, CRF levels in this brain region are increased in alcohol-dependent animals. Furthermore, stimulation of NPY activity in this brain structure suppresses anxiety-like behavior (Thorsell et al. 2007) and dependence-induced increases in alcohol drinking (Gilpin et al. 2008*a*). The anatomical distributions of CRF and NPY are highly overlapping, suggesting that one might serve as a “buffer” for the effects of the other.

#### Emerging Stress-Related Targets

Numerous other stress-related systems exist that may be important in the development of alcohol dependence, including those involving norepinephrine, orexin (hypocretin), vasopressin, dynorphin, nociceptin (orphanin FQ), neuropeptide-S, and neurokinin; an extensive overview of these systems can be found elsewhere (Koob 2008). Notable among these, recent work (George et al. 2008) has identified neurokinin-1 and its receptors as potential targets for the pharmacological treatment of alcoholism. That study found that complete (but not partial) genetic knockout of neurokinin-1 receptors suppressed alcohol drinking in mice. Based on these results, pharmacological and neuroimaging approaches were used to demonstrate that antagonism of neurokinin-1 receptors reduces craving and neuroendocrine responses^6^ to alcohol-related cues and negative-affective images in human alcoholics (George et al. 2008). This study provides an excellent example of the translational potential of basic research.

## Late Stages of Alcohol Dependence

### Neurodegeneration

Chronic exposure to high doses of alcohol can result in profound changes in the morphology, proliferation, and survival of neurons. For example, new neurons normally are constantly generated from neural stem cells throughout the life of an organism. In alcohol binge-drinking rats, however, both the proliferation of neural stem cells and the survival of neurons produced from the stem cells during alcohol exposure are decreased (Nixon and Crews 2002). Imaging studies also have revealed substantial reductions in the volumes of many brain structures in human alcoholics, particularly the prefrontal cortex and cerebellum, although prolonged periods of abstinence appear to promote at least partial recovery of these structural deficits (for a review, see Sullivan and Pfefferbaum 2005). The prefrontal cortex and, particularly, the orbitofrontal cortex^7^ have central roles in executive functions, such as decisionmaking. Accordingly, deficits in these brain areas may impact motivational circuits, impairing the ability of the organism to inhibit impulsive behavior and thereby further contributing to pathological drug-seeking behavior (Jentsch and Taylor 1999). More recently, imaging techniques were used to show that alcohol-dependent humans have smaller amygdala volumes than nondependent individuals and that smaller amygdala volume in alcohol-dependent humans is predictive of subsequent alcohol relapse (Wrase et al. 2008). This is an area of burgeoning research exploring the development, maintenance, and relapse to alcoholism in both preclinical and clinical studies.

### Protracted Abstinence and Relapse

Abstinent human alcoholics typically relapse to alcohol drinking after acute withdrawal symptoms have subsided. The resilience of relapse behavior and, presumably, the alcohol craving that underlies it is highlighted by the observation that rodents given long-term free-choice alcohol access exhibit an alcohol deprivation effect after prolonged periods (up to 9 months) of imposed abstinence (Wolffgramm and Heyne 1995). Unfortunately, such longitudinal studies are not practical for high-throughput research. Accordingly, researchers more recently have started to condense the time scale required for such analysis by using specific procedures to induce dependence more rapidly (e.g., by exposing the animals to alcohol vapor). Chronic alcohol vapor inhalation results in enhanced alcohol-reinforced behavior that lasts well beyond the dissipation of acute withdrawal symptoms (Gilpin et al. 2008*b*; Roberts et al. 2000*a*; Sommer et al. 2008). Similarly, this approach leads to increased anxiety-like behavior in rodents that persists many weeks into abstinence (Zhao et al. 2007) and can be reinstated with exposure to a mild stressor (Valdez et al. 2002). One hypothesis is that this negative emotional state contributes to relapse behavior.

Another approach for examining relapse behavior uses reinstatement models. In these models, animals first are trained to respond (e.g., press a lever) for alcohol in an operant situation. That behavior then is extinguished—that is, the animal no longer receives alcohol for pressing a lever until the animal no longer attempts to press the lever at all. Researchers can then examine the propensity of animals to relapse to alcohol-seeking behavior under three conditions that mimic the human situation: (1) following administration of a small “priming” dose of alcohol, (2) following exposure to environmental cues previously associated with alcohol, and (3) following exposure to stressors. Each of these three reinstatement models can be inhibited by different compounds (i.e., compounds that have different pharmacological profiles), indicating that they are mediated by different neural circuits. For example, one elegant experiment showed that stressors and alcohol-paired cues, both individually and additively, reinstated previously extinguished responding on an alcohol-paired lever; however, the pharmacological basis of each situation was unique. Thus, the opioid antagonist naltrexone, but not a CRF receptor antagonist, blocked cue-induced reinstatement. Conversely, a CRF receptor antagonist, but not naltrexone, blocked stress-induced reinstatement. Finally, naltrexone and CRF receptor antagonists partially blocked the additive effect of stress and alcohol cues when administered individually but completely blocked the additive effect when administered together (Liu and Weiss 2002). These findings suggest that whereas stress-induced relapse may involve the CRF system, alcohol cue-induced relapse may be mediated by the endogenous opioid system. These observations may have important implications for the development of pharmacological therapies to prevent relapse in human alcoholics.

## Conclusions

Alcohol dependence is a debilitating disease that worsens over time. New technologies are being combined with traditional approaches to identify and track the critical neural circuits in the transition from alcohol use and abuse to dependence. Substance dependence on alcohol, or alcoholism, is defined by neuroplasticity that is responsible for phenomena such as sensitization, tolerance, and withdrawal as well as for neuron survival, all of which contribute to the development and maintenance of the disorder. In addition to the extant literature on the importance of brain reward circuits in the development of alcohol dependence, recent research has focused on a new contingent of neural systems that play central roles in the regulation of stress and anxiety as well as mediate executive functions. This joint focus on brain arousal, reward, and stress systems, along with the integration of new technologies in the field, is accelerating our understanding of the components of alcohol dependence and contributing to the development of new treatment strategies.

## Figures and Tables

**Table:** Summary of Neurobiological Mechanisms of Alcohol During the Phases of the Addiction Cycle Dominated by Positive Reinforcement Versus Negative Reinforcement

**“Light Side” of Addiction: Positive Reinforcement**					
	**Baseline alcohol self-administration**	**“Binge”-like alcohol self-administration**	**Progressive ratio/second-order reinforcement schedules**	**Alcohol priming–induced reinstatement**	**Alcohol-conditioned cue-induced reinstatement**

Dopamine antagonist	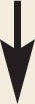	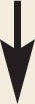			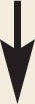
Opioid antagonist	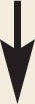	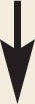	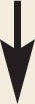	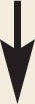	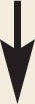
**“Dark Side” of Addiction: Negative Reinforcement**					
		**Baseline alcohol self-administration or place preference**	**Withdrawal-induced anxiety-like or aversive responses**	**Dependence-induced increases in self-administration**	**Stress-induced reinstatement**

Corticotropin-releasing factor antagonist		**—**	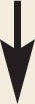	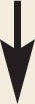	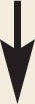
Neuropeptide Y		**—**	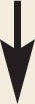	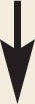	

NOTE: —, No Effect; Blank Entries Indicate Not Tested.

## Footnotes

1 For a definition of this and other technical terms, see the Glossary, pp. 279–283.

2 More recently, incentive motivation also has been termed “incentive sensitization” or “incentive salience.”

3 GABA_A_ receptors consist of several proteins (subunits). There are several types of GABA_A_ subunits, and the subunit composition of the receptors differs among different brain regions and may change in response to environmental changes.

4 Because alcohol normally reduces glutamate activity, the brain adapts to chronic alcohol exposure and maintains a “normal” state by increasing glutamate activity. When alcohol is withdrawn, heightened functionality of glutamate receptors makes neurons excessively sensitive to excitatory glutamate signals, resulting in hyperexcitability.

5 One mechanism by which electrochemical signal transmission between neurons is terminated is by reuptake of the neurotransmitter into the signal-transmitting cell. When excess neurotransmitter remains in the synapse, receptors on the presynaptic terminal are activated to prevent the release of more neurotransmitter into the synapse. Some drugs target these presynaptic receptors by blocking this “termination” signal.

6 A neuroendocrine response is the release of hormones into the bloodstream in response to a neural stimulus.

7 The prefrontal cortex is the outer layer of the brain at the front of the head. It can be divided into several regions, one of which is the orbitofrontal cortex. The prefrontal cortex is involved in high-level cognitive and executive functions, such as planning complex cognitive behaviors, decisionmaking, and moderating correct social behavior.
